# Motivations, facilitators and barriers to accessing hepatitis C treatment among people who inject drugs in two South African cities

**DOI:** 10.1186/s12954-020-00382-3

**Published:** 2020-06-10

**Authors:** Anna Versfeld, Angela McBride, Andrew Scheibe, C. Wendy Spearman

**Affiliations:** 1 Department of Anthropology, University of Cape Town, Cape Town, South Africa; 2 TB HIV Care, 11 Adderley Street, Cape Town, 8001 South Africa; 3 South African Network of People Who Use Drugs, 34 Constantia Road, Wynberg, 7800 South Africa; 4 Department of Family Medicine, University of Pretoria, Pretoria, South Africa; 5 Division of Hepatology, Department of Medicine, University of Cape Town, Cape Town, South Africa; 6 Groote Schuur Hospital, Cape Town, South Africa

**Keywords:** Hepatitis C treatment, HCV, People who inject drugs, Motivators, Facilitators, Barriers, South Africa

## Abstract

**Background:**

Treatment of hepatitis C (HCV) among people who inject drugs (PWID) is a critical component of efforts to eliminate viral hepatitis. A recent study found high HCV prevalence among PWID in two cities, Pretoria (84%) and Cape Town (44%). Very few (< 5%) HCV-infected individuals attended follow-up appointments. This sub-study explores differences between stated desire for cure and appointment attendance in light of perceived facilitators and barriers to HCV treatment and care access among PWID.

**Method:**

Two sets of semi-structured interviews were implemented in a group of HCV-infected participants opportunistically sampled and recruited at harm reduction service sites. Initial interviews, conducted before the planned hospital appointment date, asked participants (*N* = 17, 9 in Pretoria and 8 in Cape Town) about past experiences of healthcare provision, plans to attend their referral appointment and perceived barriers and facilitators to seeking hepatitis treatment. Second interviews (*n* = 9, 4 in Pretoria, 5 in Cape Town), conducted after the planned referral appointment date, asked about appointment attendance and treatment experience. Trained social scientists with experience with PWID conducted the interviews which were recorded in detailed written notes. Data was thematically analysed in NVivo 11.

**Results:**

Despite routine experiences of being stigmatised by the healthcare system in the past, most participants (*n* = 16, 94%) indicated a desire to attend their appointments. Attendance motivators included the desire to be cured, fear of dying and the wish to assist the research project. Perceived barriers to appointment attendance included fear of again experiencing stigmatisation and concerns about waiting periods and drug withdrawal. Perceived facilitators included the knowledge they would be treated quickly, and with respect and access to opioid substitution therapy. In the end, very few participants (*n* = 5) went to their appointment. Actual barriers to attendance included lack of finances, lack of urgency and forgetting and fatalism about dying.

**Conclusions:**

South Africa can learn from other countries implementing HCV treatment for PWID. Successful linkage to care will require accessible, sensitive services where waiting time is limited. Psychosocial support prior to initiating referrals that focuses on building and maintaining a sense of self-worth and emphasising that delayed treatment hampers health outcomes is needed.

## Background

South Africa has a large-scale national public healthcare system that provides prevention, care and treatment to the high proportion of the population affected by HIV. This progressive and extensive state approach, however, has not extended to people who inject drugs (PWID) despite their increased risk of contracting HIV. Criminalisation of drug use, a national reluctance to fully embrace harm reduction and high levels of stigma towards PWID [1], manifest in an absence of state-provided harm reduction services and frequently substandard healthcare provision when PWID attempt to access public services [2, 3]. In 2017, the largest (HIV-focused) needle and syringe programmes were run through non-governmental organisations (TB HIV Care and Out Well-Being) and were funded by international funders,^1^sometimes against active obstruction from state institutions. It is therefore not surprising that despite the country committing to the World Health Organization (WHO) target of eliminating viral hepatitis by 2030 [4], and despite international recognition that approximately a third of new HCV infections result from injecting drug use [5], there has been very little attention to injecting drug use as a mode of HCV transmission. Although treatment via specialist facilities is available in some cities, barriers to access are high, and there are no government services that routinely provide community-based testing and treatment for viral hepatitis for PWID. The Viral Hepatitis Initiative for Key Populations in South Africa (the Viral Hepatitis Initiative) was a cross-sectional survey implemented in seven South African cities (recruiting PWID in three of these) between August 2016 and October 2017. This survey included testing for HCV, HBV surface antigen and HIV among PWID who were opportunistically sampled as they accessed health services and it confirmed a high overall HCV seroprevalence of 55% among PWID (84% in Pretoria, 44% in Cape Town and 35% in Durban) [6].^2^ Unlike HIV, HCV infection is curable and treatment is becoming easier with direct acting antiviral therapy (DAAs) [7]. In South Africa, DAAs for HCV are not registered but can be accessed after obtaining a Section 21 approval from the South African Health Products Regulatory Authority (SAHPRA). Accessed DAAs are either self-funded, funded by medical aids or state-funded through motivation at academic teaching hospitals.

HCV treatment for PWID is recommended in international guidelines [8], and treatment adherence and cure rates amongst PWID accessing DAAs, at > 90%, are acceptable [9, 10]. Yet, globally, treatment uptake remains unacceptably low [10–12]. In the Viral Hepatitis Initiative, people who tested HCV antibody positive were offered linkage to HCV care. This was unescorted, and the nature of the services on offer differed depending on what was available: In Pretoria, referrals were made to Tshwane District hospital for liver monitoring and support. In Cape Town, HCV-infected individuals received referrals and appointment dates for the Groote Schuur Hospital Liver Clinic, which is affiliated to the University of Cape Town and the only facility that routinely applied for DAAs to treat HCV-infected individuals using the Section 21 avenue.

Early on in the Viral Hepatitis Initiative, implementing staff and referral hospital teams noticed that in both Pretoria and Cape Town newly diagnosed individuals (almost exclusively PWID) would indicate a desire for follow-up support and treatment but then would not attend initial or rescheduled appointments. Low HCV treatment uptake among PWID in a range of contexts [10, 11] is linked to various barriers, including individual, social and health system level barriers [13]. However, there is no literature that speaks to this in the South African context. This study, a sub-study of the Viral Hepatitis Initiative, sought to understand barriers and facilitators to care access.

## Method

The Viral Hepatitis Initiative took place in sites that routinely provided harm reduction and HIV prevention services for PWID. In two of the sites (Pretoria and Cape Town), PWID who had tested HCV positive on point of care tests were invited to participate in this study, which was structured into two sets of semi-structured qualitative interviews. The first interview, implemented before the planned hospital appointment date, asked participants to reflect on past experiences of healthcare provision, plans to attend their referral appointment and perceived facilitators and barriers to seeking viral hepatitis treatment. The second interview, implemented after the planned appointment date, asked participants about appointment attendance. Those that had attended were then asked about their treatment experience. Those that had not attended were asked about their reasons for non-attendance. This line of questioning was based on the discrepancies service providers had reported between initial responses to a positive HCV diagnosis and follow-up health-seeking behaviour.

All interviews occurred between July and December 2017. The time between the interviews was dependent on referral appointment date, which at the specialist treatment centre in Cape Town was sometimes months in advance. In some cases, first and second interviews were a week apart; in other cases, they were months apart. However, all second interviews were conducted within 6 weeks of the referral appointment date.

Two trained social scientists conducted the interviews. AV, the Cape Town interviewer, is a medical anthropologist who, at the time of the research, had 7 years of experience conducting research with people who use drugs, including in the implementing organisation. AM, also a trained anthropologist and the Pretoria interviewer, had worked as a counsellor in the implementing organisation for 2 years at the time of the research. Both interviewers were involved in the parent study: AV was in the management team and worked across all study sites with staff teams; AM was an implementing team member in Pretoria.

In both sites, recruitment and sampling for the first interview was opportunistic. Participants were approached if they were eligible and at the fixed service provision sites while the researchers were available. In Cape Town, staff who had been involved in the parent study (and were privy to individuals’ HCV status) informed participants about the potential study and linked interested participants with AV on days that AV was at the site. In Pretoria, AM approached potential participants herself. Inclusion was entirely based on who arrived first. The result was a sample that was largely reflective of the parent study. The majority (*n* = 15) were male; the lower number of women (*n* = 2) reflects the lower number of women participating in the parent project in the two included cities (15%). The age of participants ranged between 29 and 45, with a mean age of 36. Four participants self-classified as black, 6 as white and 7 as of mixed ancestory. The slightly (10%) higher proportion of white participants in this study compared to the parent study reflects the demographic profile of people accessing the fixed services, rather than mobile services in Pretoria. Eleven (65%) of the participants were sleeping rough.

Participation was not remunerated. As an add-on study to a parent study that was incorporated into service provision and therefore not remunerated, there was no available budget. In the context of people using drugs for whom time is often directly linked to earnings that can stave off withdrawal, this may have shaped who was willing to talk to us. This study included 17 participants in the first round (9 in Pretoria and 8 in Cape Town). A subset of these took part in the second round of interviews (9 in total, 4 in Pretoria, 5 in Cape Town). This met our set recruitment targets of between 12 and 20 participants in initial interviews and between 8 and 15 in the second interviews. In Pretoria, we had no inclusion refusals (in Cape Town, we did not ask recruitment staff to document these refusals). The lack of incentives may, in fact, have had the positive impact of meaning that the people who spoke to us likely did so out of genuine concern for improving the health conditions they face daily.

Eligibility required participants to indicate their willingness to participate in both interviews, but as indicated in our targets, a drop off between interview rounds was expected. This was because participants did not all have contact details or regular life patterns. Inclusion in the second interview either relied on their actively seeking out the researchers to make an arrangement or their being present, willing and able to talk at a time the researchers were available on site during the research period. In Pretoria, all of those who were not in the second interview returned to talk to AM but after we had closed the recruitment period. In Cape Town, the two people who did not participate in the second interviews both indicated that they were busy at the requested second interview time.

Our work was undoubtedly affected by the extent of relationships with the participants. AV knew two participants well from having trained them on qualitative research methods, but the rest she either did not know or only knew by sight. AM knew all the participants from having interacted with them during service provision. This may have meant that participants were more likely to provide answers they thought the interviewer wanted to hear. At the same time, people who use drugs have little incentive to explain life worlds to people they do not know [14, 15] and it may be that the candidness of the responses we received was to some extent related to a level of trust built on long-standing relationships. In recruitment and interview processes, it was emphasised to the respondents that their answers would in no way affect their access to services.

The interview location was determined by where participants indicated they were most comfortable. In Cape Town, where the facility was more crowded, most (*n* = 10) were conducted outside, in a quiet corner of a public square close to the drop-in centre, outside of earshot of other people. The rest (*n* = 4) were conducted in-facility in a clinical or counselling room behind closed doors. In Pretoria, where there was less immediate public space and more privacy within the facility, most interviews were conducted in a counselling room (*n* = 7) and when this was occupied, the boardroom (*n* = 4), with only 2 conducted outside. Interview time length ranged between 20 and 45 min, with most—across both interviews—taking approximately 30 min.

Voice recorders were not used because researcher desires to keep the conversations as pressure-free as possible and concerns about confidentiality and security. The interview sites were sites of frequent theft of valuable items, and before this study, one of AV’s voice-recorders was stolen during a research process. Interviews were, therefore, recorded through detailed ethnographic written notes, which included—amongst other things—information about interview location, affect and past engagement with the participants. These notes were securely stored off-site and typed up as soon as possible, mostly within hours of the interview completion. Where direct quotes were written down word-for-word during the interview process, these have been included in the text, though challenges in finding time with participants meant they were not member checked. Both interviewing researchers analysed the interview write ups and separately developed coding matrices until saturation was reached. The researchers then compared matrices and developed a unified framework for all the interviews. These were then coded in NVivo 11 by one researcher (AV) and cross-checked by the second (AM).

Research was approved by the University of Cape Town Human Research Ethics Committee (reference number 320/2017). Informed consent was obtained from all participants. Pseudonyms are used in this paper, and identifying features of participants have been withheld.

## Results

### Desire to attend appointments

All but one of the participants (*n* = 16, 94%) indicated, mostly emphatically, that they would attend their initial hospital appointments. They reported concerns about their health and the desire to be cured. This was particularly pressing for the four participants that indicated they were experiencing symptoms that they ascribed to hepatitis (mostly lethargy), but these concerns were also expressed by those not experiencing symptoms. As Taariq (male, 30, Cape Town) said, “I care about my health. I have kids and I want to be healthy for them…I want to be cured.”

The flipside to this desire for cure was, for some, the fear of illness and dying. Jesse (male, 44, Cape Town) explained that he was planning to attend his appointment “because I don’t want to die...” On being asked whether he thought hepatitis was going to result in his death he said, “Yes. Nathalie Cole died of hepatitis last year, and she was rich. If a rich woman died, what is my chance [of surviving]?” Shafiek (male, 31, Cape Town) explained that he did not want to “be like all those others who go [die] so easily”. He explained that he had heard of four people who had tested positive for hepatitis dying recently, two were his friends. He (rightly or wrongly) attributed these deaths to hepatitis.

The intention to attend appointments was also shaped by desire to assist the implementing teams (through gathering information) and/or to see whether healthcare systems had improved since the project team had engaged with staff at the referral centres. Edward (male, 35, Pretoria) said, on enquiry if he would attend the facility he was referred to, that he would attend for the research and that he would “even take a notebook with”, although previously he had stated he would not normally go, even if methadone was made available to him. Wolfgang (male, 45, Pretoria) also stated he would visit the hospital for the sake of the research as well as to see if the processes had changed, whether the queues were shorter, if the hospital provides HCV treatment and how people were being treated by medical staff.

### Past experiences of stigma

The voiced desire to attend appointments in all but one participant (who expressed ambivalence) was despite widely reported negative past experiences in the healthcare sector. These experiences included being blamed for poor health, being made to feel ashamed for injecting drugs, being told that people who use drugs are taking attention away from deserving people and being made to wait. For example, one participant, Taariq described his last interaction with the public healthcare system: he had gone to an emergency unit because he had unintentionally injected heroin into the muscle of his arm and not into his vein, resulting in extreme swelling. He said he was left to wait an entire night before seeing a doctor who “went off” when he revealed the cause of his injury. He explained how the doctor shamed him by saying, “there are other people out there with real sicknesses, and now I must sit here with you”. Taariq said that the worst thing was that the doctor did not even touch his arm to assess the severity of the situation and told him to go away and come back if it got worse so that they could amputate his arm.

Participants also described privacy and confidentiality breaches. Martin (male, 48, Cape Town) described a doctor revealing his hepatitis status in front of other people in a waiting room, and Jesse described a nurse denying him a requested medical certificate for work, telling him, in front of a corridor full of people, that he should rather go “hustle” (make money/beg) for money in the main road—an activity associated with drug use. As Greg (male, 30, Pretoria) summed up “ [the healthcare staff] are not helping you, they’re oppressing you”.

Participants also reported being ignored or made to wait excessively—beyond what was generally expected within the public healthcare system—as a form of active punishment for people who used drugs. As Amina, (female, 31, Cape Town) described, “If they hear you are using you must wait until there is someone available, *if* there is someone.” Amina further described her interaction with clinic staff saying, “The lady saw on my arm I was using, she didn’t want to communicate with me” and going on to say that she was left waiting to be served until last, despite other people having arrived after her. “Being an addict at the hospital they treat you unfair…it is like your body is nothing,” she said.

### Perceived barriers and facilitators to care access

None of the participants had previously accessed hepatitis care, but all had concerns about how they would be treated in the healthcare system. Gary (male, 30, Pretoria) clearly stated that fear about how he would be treated was a potential barrier to attending his appointment. He stated that now that he was maintaining his personal hygiene, he would visit the hospital but when he was living on the street and unable to access ablution facilities, he would not “waste his time” because he would leave without being attended to. Roger (male, 29, Cape Town) said he was concerned because the way people looked at him made him feel like a “freak”. However, despite the many past experiences of stigmatised treatment, most people did not identify these as inhibitors of accessing future treatment. Rather, what emerged for most participants as pressing was withdrawal, and fear thereof. As Roger (male, 29, Cape Town) explained, “every addict fears cold turkey”, then correcting himself, he said that what every drug user fears is not having drugs available and leaving the place where they can reliably make money. Johan (male, 45, Cape Town) said the only thing that would stop him attending his appointment would be if he “didn’t have a fix laid out”.

A fear of waiting was closely associated with the concerns of withdrawal. Shafiek said that he had never been to a public healthcare facility because he knew waiting was required, “I’m an addict. I don’t have the time and patience to wait,” he explained. Roger said, “I hate sitting long. I get irritable. A heroin addict hates sitting long.” Wolfgang (male, 45, Pretoria) explained that people who inject drugs are impatient because of the need to generate income but also indicated that immediate treatment was not a reasonable expectation to have. These issues (time required and fear of withdrawal) overshadowed other possible imagined reasons for not going to an appointment, which included possible life crises, such as a child being extremely sick (*n* = 2), not having transport money (*n* = 1) and work (*n* = 1).

When asked what would assist appointment attendance, almost half the participants (*n* = 8) said that access to either opioid substitution therapy or to enough drugs for the day would increase the likelihood of them visiting the hospital. Six participants indicated that knowing they would receive treatment that was professional and of high quality, or at least standard to that received by other people, would increase their likelihood of visiting the facility. As Jo (male, 25 Pretoria) stated “everyone just wants to be treated the same, equal”. Five participants also indicated that knowing that they would be quickly attended to would make a difference, noting that these extended waiting periods fed into the time they would be using to make money. This was explained by Wolfgang (male, 45, Pretoria) who said that PWIDs do not have the time to wait 3 or 4 h to be treated because that they need to “zula” (hustle/make money/beg).

### Reported reasons for non-attendance

Only five of all the participants (29%) attended their first appointments at the referred hospitals (3 in Cape Town, 2 in Pretoria). We divide the reasons provided for lack of attendance into three categories: lack of finances, lack of urgency, and fatalism. Lack of finances was the key reason for non-attendance participants provided. This was despite the fact that most people had confidently asserted that they would make sure they had enough money through generating it in the days running up to the appointment. Having no money was identified as being linked to the need for a daily dose so as to avoid withdrawal, transportation to and from the healthcare facility, and basic needs, such as food for the day. As Roger explained, “I didn’t have money and I wasn’t going to go to town with no wake-up stuff [drugs]”. Damon (male, 35, Pretoria) said that without money for transport he was unwilling to walk to the facility in the cold, with the expectation that he would more than likely be treated badly by the facility’s staff. Mary (female, 43, Pretoria) reiterated the above when she stated “[If] I spend the whole day at the hospital. Who’s going to make a plan for food that evening?”

Non-attendance was also simply described in terms of forgetting. Brandon (male, 25, Cape Town) indicated that, “I wanted to go, then I forgot my appointment date. The date was only in October and in that time I was drugging a lot.” Roger similarly said, “It slipped my mind when I went home, only on the day of my appointment I realised.” Forgetting was perhaps supported by a lack of sense of urgency to attend the appointment. Johan (male, 45, Cape Town) explained that getting treated was simply not at the top of his priority list. One participant went away to an inpatient drug rehabilitation centre at the time of his appointment. Another was able to go but said that, “I know that the hepatitis virus attacks you in a few years’ time, that’s why I’m taking it a bit easy…” He said that he knew that hepatitis was a slow-moving virus and this meant he did not feel the need to respond immediately. Damon (male, 35, Pretoria) had a similar mindset concerning the lack of urgency, but this was also linked to a lack of available treatment by stating “I don’t have to go now, I can go anytime, but I’ll go later when I find out what they’ll do about it [HCV]”. This lack of urgency, it seems, was partly supported by the fact that participants seemed to have insufficient information about what a positive diagnosis meant and the impact of HCV on one’s health. Wolfgang (male, 45, Pretoria) stated that they [PWID community] do not realise how dangerous HCV is and that there needs to be more emphasis on education on the subject.

Knowing the severity of the virus was not, however, necessarily enough to motivate attendance in the face of a lack of sense of self-worth and fatalism described by a number of participants. Jesse (male, 44, Cape Town) who had previously been diagnosed and not attended appointments said, “I wasn’t really giving a shit if I live or I die. Death didn’t seem that unappealing.” Similarly, another participant explained that he did not care what happened to him, saying, “you don’t give a f*&% just let nature take its course”. Greg (30, male, Pretoria) questioned his right to care, saying, “Who am I? I brought this to myself”, and another participant said there was no point getting treatment if he was simply going to get re-infected.

### Reported attendance reasons and experiences

Various motivating factors were described by those who did attend their appointments. These included trusting the referral team and a reduction in “self-stigma” at having learned how common HCV is in PWID (Jesse, 44, Cape Town), active persuasion on the day of the appointment by staff members at the harm reduction facility (Taariq, male, 30, Cape Town) and the support of a peer navigator (Wolfgang, male, 45, Pretoria). Edward (male, 35, Pretoria) said that his attendance was not for himself but rather to support the research project. Attendance experiences varied. Both the Cape Town participants described the sense of value they felt when it was clear that the healthcare staff were expecting them and did not stigmatise them for their use, but rather encouraged them to stay and wait precisely because they were PWID. In Pretoria, Edward said that he waited the whole day, was chided for his drug use loudly in front of other waiting patients, suffered serious withdrawal and eventually left at the end of the day without having received assistance. Wolfgang, in contrast, was efficiently moved through the system with support of the peer navigator and described his appointment as acceptable, if disappointing due to a lack of available treatment.

## Discussion

The literature indicates a wide range of barriers to care for HCV-infected PWID. These include reluctance to engage with healthcare providers due to feelings of shame about drug use [16] and prior experiences of stigma and discrimination in the healthcare system [17–19]. Related to this are high levels of patient mistrust of the healthcare system [16, 20] and fear of the treatment process, often fuelled by stories of the side effects of interferon-based therapy [16, 20–23]. Low levels of perceived need for treatment [24] and/or a corresponding lack of a sense of urgency, especially for those who are asymptomatic [16, 20]^,^ and other health priorities [22] all negatively impact on treatment seeking. Practically, barriers such as distance from the place of treatment to their place of residence, appointment time [21] and for countries where this is a requirement, lack of insurance and treatment costs [24] further undermine engagement in treatment. Provider-level factors also contribute negatively. Medical staff are often reluctant to treat PWID, especially if they are still injecting [25–27], for reasons such as a mistrust in the person to adhere to treatment because they use drugs, as well as concerns about costs, co-morbidities and reinfection [25, 26, 28, 29]. These barriers are all compounded by the criminalisation of drug use [10, 30].

Our research, the first of this nature to be conducted in South Africa, echoes many of the findings from other parts of the world. Most notably, participants reported past experiences of stigma and high levels of mistrust of the healthcare system, low levels of urgency and practical barriers to appointment attendance. Our research was, however, structured differently to other published literature in that we explored reported self-expectations of behaviour and anticipated barriers as well as actual behaviour and experienced barriers. This reveals a mismatch between the participants stated intention (and perceived capacity) to attend their appointments and their actual attendance. In initial interviews, individuals seemed confident that past experiences of stigma would not affect their attendance and though they recognised lack of resources as a potential barrier to attendance, they also reported confidence that they would manage to muster enough resources to get to their appointments and avoid withdrawal. This did not prove to be the case, and most participants did not attend their appointments. It may be that in the first interview participants simply wanted to assure the researchers that they were enacting ideal behaviours as clients of the harm reduction services they were accessing. Our impression was, however, that they were assuring themselves that they would act in the best interests of their own health in the future. This motivation, however, waned in the interim between the interview and the appointment date, for some this was overridden by a combination of lack of urgency, low sense of self-worth and lack of conviction that life is worth living.

The inaccuracy in envisioning, or verbalising, potential barriers to treatment access contrasted with the participants’ ability to conceptualise potential facilitators. It was the lack of opioid substitution therapy, or sufficient resources to facilitate transport and avoid withdrawal while waiting for services, that were reported most frequently to be the cause of appointment non-attendance. Our work confirms that the key facilitators to care access noted elsewhere—access to opioid substitution therapy [12] continuity of care [23] and accessible, integrated, treatment facilities [30, 31]—need to be addressed to enable cure and reduce risks of re-infection. Our interviews with people who did attend their appointments all indicated the importance of established relationships of trust as a key motivating factor for ongoing treatment seeking. They further showed how being well-treated by healthcare provider fostered an important sense of self-worth. Psychosocial support processes that build a sense of self-worth and the right to care as could, as others suggest, be provided by peer networks [29].

To the best of our knowledge, all the participants who did attend their appointments participated in the second interview. Furthermore, the percentage of our participants (at 29%) who attended their appointment is far higher that the percentage of all the referred participants of the larger study that attended their appointments (< 1%). This indicates a positive relationship between appointment attendance and willingness to engage in the second interview, which is likely bi-directional: the fact of the forthcoming second interview supported clinic appointments, and clinic appointment attendance encouraged second interview participation. This suggests the importance of follow-up of people referred, as well as the positive impact rapport has on the willingness to engage and participate.

The study was subject to a number of limitations. The lack of remuneration was likely to have shaped recruitment and affected participation in second interviews. The small sample size and qualitative nature of this study limits the generalisability of the findings, and the relationship of the researchers to the parent study may have influenced the ways in which participants provided answers. We recognise that other factors may have arisen had further interviews been conducted. Additional insights would also have been obtained if liver-related symptoms were captured; however, the fact that not one of the four (3 men, 1 woman) that indicated they were experiencing symptoms attended their follow-up appointments indicates that that this was not necessarily correlated. Greater inclusion of women would likely have highlighted additional issues. Women who use drugs in South Africa [2] and beyond face substantial additional barriers to healthcare access, including HCV treatment [32]. Further research related to the challenges women who use drugs face when attempting to access care is needed. Finally, we acknowledge that not using voice recordings may be seen to undermine the rigour of the data due to researcher bias and resulted in the limited inclusion of direct quotes. Given that the researchers are anthropologists, experienced in the field and trained in reflexive and careful capture of social interaction, we suggest that the detailed interview notes that were developed provided a nuanced picture of the factors at play.

## Conclusion

HCV treatment can be affordable and is effective and well tolerated by PWID. However, eliminating viral hepatitis will require a concerted effort in which PWID are treated as worthy individuals and supported in recognising themselves as such. Hepatitis services need to be accessible and quick to reduce the financial and time costs of attendance. They further need to be consistently friendly, as experiences of stigma travel. As far as possible, services should be integrated into holistic care, inclusive of opioid substitution therapy. Services should also acknowledge and seek to overcome the fact that verbal commitments to access care may not align with actual capacities and that a desire to attend services may be undermined by low self-worth. Psychosocial support prior to initiating referrals that focuses on building and maintaining a sense of self-worth and emphasising that delayed treatment hampers health outcomes is needed.

## Data Availability

It is not possible to fully de-identify fieldnotes and interview write-ups in order to fully ensure participant confidentiality. These will therefore not be made available on a public repository. However, de-identified sections of data will be made available on request.

## Abbreviations

DAA: Direct-acting antiviral
HCV: Hepatitis C virus
OST: Opioid substitution therapy
PWID: People who inject drugs
WHO: World Health Organization

## Footnotes

1 The Step Up Project, which seeks to provide HIV prevention services to people who inject drugs, was initiated in 2015 in three cities (Cape Town, Pretoria and Durban) and extended to a fourth in 2017.

2 The Viral Hepatitis Initiative was implemented by TB HIV Care in Partnership with OUT Well-Being, Anova Health Institute, the University of Cape Town’s Division of Hepatology and the National Institute of Communicable Diseases.
